# Vascular calcification in skin and subcutaneous tissue in patients with chronic and end-stage kidney disease

**DOI:** 10.1186/s12882-020-01928-0

**Published:** 2020-07-16

**Authors:** Irene Ruderman, Tim D. Hewitson, Edward R. Smith, Stephen G. Holt, Belinda Wigg, Nigel D. Toussaint

**Affiliations:** 1 Department of Nephrology, The Royal Melbourne Hospital, 300 Grattan St, Parkville, Victoria 3050 Australia; 2 Department of Medicine (RMH), The University of Melbourne, Parkville, Victoria Australia

**Keywords:** Vascular calcification, Chronic kidney disease, End-stage kidney disease, Calcific uraemic arteriolopathy

## Abstract

**Background:**

Vascular calcification (VC) is well described in large- and medium-sized vessels in patients with chronic kidney disease (CKD), especially in those with end-stage kidney disease (ESKD) on dialysis. Medial calcification is particularly prevalent in this population and contributes to arterial stiffness and increased cardiovascular mortality and morbidity. Apart from in the setting of calciphylaxis, few studies have assessed skin and subcutaneous calcification and associations with abnormalities of bone and mineral metabolism in patients with CKD.

**Methods:**

We performed a single-centre observational study to evaluate incisional skin tissue samples from three anatomical sites in patients with different stages of CKD undergoing elective surgery. We compared these samples to skin samples of a control cohort without CKD. Staining for calcification was performed with von Kossa method. A subgroup of skin samples were assessed by RT-PCR for upregulation of pro-calcific gene transcripts for tissue non-specific alkaline phosphatase (*TNAP)* and Runt-related transcription factor 2 (*RUNX2*).

**Results:**

Forty-five patients were evaluated, 34 with CKD (including ESKD) and 11 control patients. VC was identified in 15 skin samples (13 CKD/ESKD and 2 controls). VC was present in the dermal and subcutaneous tissues of the neck, abdomen and arm samples. Two different histological types of VC were identified: speckled medial calcification and internal elastic lamina calcification. Presence of perieccrine calcification was identified in 14 samples, 10 with concurrent VC. There were no significant differences in serum parathyroid hormone, phosphate or calcium in patients with or without VC. Expression of *TNAP* or *RUNX2* was not increased in samples from patients with ESKD or those with histological evidence of calcification.

**Conclusion:**

This study reports the novel finding of dermal and subcutaneous calcification in multiple anatomical locations in 38% of patients with advanced CKD/ESKD undergoing elective surgery but free from calciphylaxis.

## Background

Vascular calcification (VC) is prevalent and well described in large- and medium-sized vessels of patients with chronic kidney disease (CKD), especially in those with end-stage kidney disease (ESKD) on dialysis [1, 2]. Patients with CKD are at higher risk of VC compared to the general population, irrespective of age and co-morbidity burden [3]. Medial calcification is particularly prevalent in the CKD population and is associated with arterial stiffening and higher cardiovascular morbidity and mortality [4, 5]. Medial arterial calcification has been identified in multiple anatomical locations in patients with ESKD, including normal breast tissue from mastectomy samples [6], as well as subcutaneous tissue of lower limb amputations [7]. In both locations, VC has been identified in vessels in the presence of other patient risk factors for calcification, including active malignancy or peripheral vascular disease.

Conversely, the presence of VC of small vessels in the skin and subcutaneous tissue has only been systemically described in ESKD patients with calciphylaxis [8], although recent studies suggest even this may not be a specific finding and VC can be present in patients with peripheral vascular disease without the presence of calciphylaxis [7]. Calciphylaxis is considered an accelerated template of widespread VC. It is characterized by severe vessel ischaemia accompanied by vessel thrombosis and surrounding panniculitis [9], ultimately resulting in painful skin lesions which typically break down to non-healing ulcers that are prone to infection, with accompanying high mortality [10, 11]. However, calciphylaxis is a rare condition and only occurs in a very small minority of patients with ESKD with approximately 3.5 cases per 1000 patient years in those receiving chronic haemodialysis therapy [12] To date, there is little information about the prevalence of calcification in skin and subcutaneous tissue in patients with advanced CKD and ESKD outside the setting of calciphylaxis, and if present, whether it is related to disordered bone and mineral metabolism.

The aim of the present study was to characterize VC changes in the skin and subcutaneous tissue of patients with CKD compared to a control group. We also aimed to evaluate associations between skin calcification and biochemical markers of deranged bone and mineral metabolism and identify any other histological features present that are typically described in calciphylaxis.

## Methods

### Patients

Between May 2017 and March 2019 patients undergoing a planned surgical procedure performed by the Nephrology Surgical Team at The Royal Melbourne Hospital were approached to participate in this single-centre cross-sectional study. Patients enrolled underwent elective surgical procedures including parathyroidectomy, hernia repair and arteriovenous fistula (AVF) formation. A fasting blood sample was collected at the time of surgical procedure. Patients over the age of 18 and able to provide informed consent were included in the study. There were no exclusion criteria.

### Ethics approval and consent to participate

The study was approved by the Melbourne Health Human Research Ethics Committee (#HREC 2017.023) and was conducted in accordance with the Declaration of Helsinki. Written informed consent was obtained for this study.

### Histology

A full-thickness incisional skin sample was collected by the operating surgeon from the surgical site. Tissue samples were fixed in neutral buffered formalin and processed to paraffin wax for histology. Serial paraffin embedded skin sections were stained with haematoxylin and eosin (H&E) and von Kossa. Cross-sections of skin and subcutaneous tissue were evaluated in their entirety.

Counting of vessels was performed on slides stained with H&E due to ease of identifying vessels. Each cross section contained epidermal, dermal and subcutaneous tissue, with tissue blocks re-cut if they did not contain all three areas. Vessel size was estimated using a graticule. Typically, arterioles ≤50 μm in diameter have either absent or partially complete internal elastic lamina (IEL) [13, 14]**.** As a result, differentiating terminal arterioles from terminal venules is very challenging, therefore all structures with a lumen were defined as vessels. Samples with greater than 10 vessels per cross section were considered to have adequate vessel number. H&E sections were examined for evidence of intimal hyperplasia, intimal thrombosis, pannicultis or hyperplasia.

Identification of calcification was performed on slides stained with von Kossa. In brief, sections were dewaxed and rehydrated before being immersed in a solution of freshly prepared 5% Aqueous Silver Nitrate. Tissue sections were placed at close range under a bench lamp and exposed to a strong light source for 30 min. They were then washed in distilled water. To remove the unreacted silver, slides were covered with 5% Sodium Thiosulphate for 2 minutes. This was rinsed in distilled water before being counter stained with Working Eosin solution for 2 minutes. For each case, the following features were graded as either present or absent: calcification of any sized vessels, presence of extra-vascular calcification, IEL calcification. Results were expressed as a % of all vessels in the section. Histologic sections of all cases were counted independently by two investigators (IR and BW) with results presented as an average of the two observers. Malignant necrotic breast tissue was used as a positive control for the von Kossa stain.

### Real-time reverse transcriptase polymerase chain reaction (RT-PCR)

Total RNA was isolated from a subgroup (13 out of 43) of patient samples stored in RNAlater solution (Invitrogen, Carlsbad, CA, USA) using the RNEeasy Fibrous Mini Kit (QIAGEN, Hilden, Germany) in accordance with manufacturer’s instructions. To minimise sampling error, triplicate 30 mg portions of tissue from each sample were processed. Quantification of RNA was performed using the Qubit 4 Fluorometer and Quant-IT RNA Assay Kit (Thermo Fisher Scientific, Waltham, MA, USA) according to the manufacturer’s instruction with 1 μg used as a template for cDNA synthesis using the iScript RT supermix kit (BioRad, Hercules, CA, USA). Quantitative real-time PCR (qRT-PCR) was performed in triplicate in a CFX96 cycler (Bio-Rad) using equal volumes of cDNA (2 μL) and the SsoAdvanced™ Universal SYBR Green Supermix (Bio-Rad) and validated PCR Prime assay primer pairs (Bio-Rad): *TNAP* (Unique Assay ID:qHsaCID0010031); *Runx2* (Unique Assay ID: qHsaCID006726); and glyceraldehyde-3-phosphate dehydrogenase (*Gapdh*) (Unique Assay ID: qHsaCED0038674). PCR conditions were set according to the manufacturer’s instructions. Melt curve analysis was performed to verify the purity and specificity of the amplicons. Threshold cycles were calculated using CFX Manager Software (Bio-Rad). The mRNA level of target genes was normalised to the house keeping gene *Gapdh*, and expressed relative to an appropriate control using the 2^(−ΔΔCt) method. Results are presented as the mean of all three samples.

### Statistical analysis

A sample size estimation for this cross-sectional study was 50 patients based on feasibility with the number of patients undergoing elective surgery at our institution over the recruitment period as well as a larger enough cohort to allow for heterogeneity of skin sample sites and different stages of CKD. All data were summarised, and results reported as mean (standard deviation [SD]) or median (inter-quartile range [IQR]) for continuous data and number (percentage, %) for categorical variables. Comparison of the values of continuous variables between groups was made using an unpaired t test or Wilcoxon signed-rank test as appropriate. Chi-squared and Fisher’s exact tests were used to investigate associations between various categorical variables. Two-tailed *P* values of < 0.05 were considered to be significant. All statistical analyses were performed using SPSS version 21.0 for Macintosh (SPPS, Chicago, IL). Graphics were created with GraphPad Prism 8 for Macintosh (la Jolla, CA, USA).

## Results

### Demographic and biochemical characteristics

Forty-five patients undergoing elective surgical procedures were enrolled in the study. Eleven patients with normal kidney function (mean eGFR 83 ± 10 mL/min/1.73m^2^) were included in the control group. Of these 11, 2 underwent donor nephrectomies, 5 hernia repairs, 1 laparoscopic cholecystectomy and 3 total thyroidectomies. Of the 34 patients with advanced CKD (mean eGFR 21 ± 0.5 ml/min/1.73m^2^) and ESKD, 20 underwent AVF creation, superficialization or ligation, 7 parathyroidectomies, 2 kidney transplant nephrectomies, 2 peritoneal dialysis catheter insertions, 2 hernia repairs and 1 total thyroidectomy.

Relevant patient comorbidities and biochemistry are summarised in Table 1. The mean age of the CKD group was 60 ± 16 years; 76% (*n* = 26) of patients were on haemodialysis, 15% (*n* = 5) on peritoneal dialysis and 9% (*n* = 3) had CKD stages 4 and 5 not on dialysis. The comorbidity burden was greater in the CKD group compared to the control group; 53% (*n* = 18) of patients were either former or current smokers, 38% (*n* = 13) had a history of diabetes, 70% (*n* = 24) had hypertension, and 12% (*n* = 4) were documented to have peripheral vascular disease. The median body mass index (BMI) of patients was in the overweight range for both the control and CKD cohort. As expected, there were significant differences in mineral biochemistry between the control and CKD cohort, with higher levels of serum parathyroid hormone (PTH) (*p* < 0.001), phosphate (*p* = 0.002) and alkaline phosphatase (ALP) (*p* = 0.03) in patients with CKD. None of the patients were receiving warfarin. Sixteen patients in the CKD group were prescribed calcitriol and 25 prescribed phosphate binders, 12 of who were on a calcium-based phosphate binder.

**Table 1:** Clinical features of study cohort

Demographics	CKD (***n*** = 34)	Control (***n*** = 11)	***P*** value
Age, years	60 ± 16	62 ± 9	0.3
Gender, male	22 (69%)	4 (36%)	
BMI (kg/m^2^)	27 [24–33]	27 [24–31]	0.8
Dialysis modality	26 HD, 5 PD, 3 CKD	n/a	
Time on dialysis (years)	4 [2.8–9.8]		
**Comorbidities**			
Smoker (current)	5 (15%)	2 (18%)	0.6
Smoker (former)	13 (38%)	2 (18%)	0.8
Diabetes	13 (38%)	2 (18%)	0.8
Hypertension	24 (70%)	2 (18%)	0.7
IHD	7 (20%)	0	
PVD	4 (12%)	0	
Parathyroidectomy	4 (12%)	0	
**Biochemical parameters**			
Corrected calcium (mmol/L)	2.3 ± 0.2	2.3 ± 0.2	0.65
Phosphate (mmol/L)	1.9 ± 0.6	1.1 ± 0.2	0.002
ALP (IU/L)	107 [81–149]	74 [64–102]	0.03
PTH (pmol/L)	62 [37–148]	4.6 [0.9–8]	< 0.001

Results are mean ± SD, median [IQR] or number (%)

*Abbreviations*: *ALP* alkaline phosphatase, *BMI* body mass index, *HD* haemodialysis, *IHD* ischaemic heart disease, *PD* peritoneal dialysis, *PTH* parathyroid hormone, *PVD* peripheral vascular disease

### Anatomical and histological features of participants

**Fig. 1:**
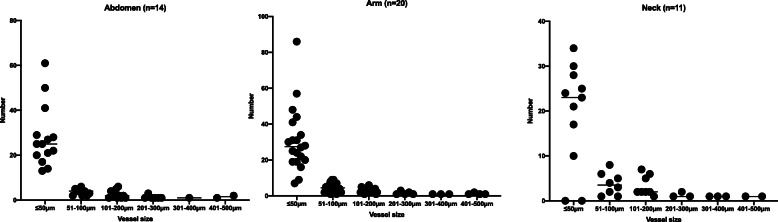
Number of vessels per vessel diameter in each patient based on anatomical location. Each dot per column represent individual patient

Anatomical location and histologic features of the skin samples are summarized in Table 2. All sections contained a minimum of 10 vessels, with a mean of 41 ± 16 and 34 ± 17 vessels per slide in the CKD/ESKD and control groups, respectively. All sections contained full thickness skin tissue including epidermis, dermis and variable amount of subcutaneous tissue depending on the anatomical location of the sample. The mean number of vessels did not differ significantly across the three anatomical locations (*p* = 0.17) or between groups. Vessels ≤50 μm in diameter accounted for 75% of total vessels counted. The largest vessel identified was 500 μm in diameter from an arm skin sample. Each sample had a mean of 5 ± 0.6 vessels ≥100 μm in diameter. Figure 1 demonstrates the number of vessels per vessel diameter in each patient based on anatomical location.

**Table 2:** Anatomical and histological features of skin tissue samples

Histologic features	CKD (***n*** = 34)	Control (***n*** = 11)
Location, number of samples		
• Neck	8	3
• Abdomen	6	8
• Arm	20	0
Vessel number, mean ± SD		
• All sites	42 ± 17	34 ± 13
• Neck	37 ± 7	47
• Abdomen	63 ± 18	39 ± 15
• Arm	37 ± 14	n/a
Vascular calcification present, number (%)	13 (38%)	2 (18%)
Perieccrine calcification, number (%)	13 (38%)	1 (9%)

Results are mean ± SD, or number (%)

### CKD/ESKD patients have high rates of VC

**Fig. 2:**
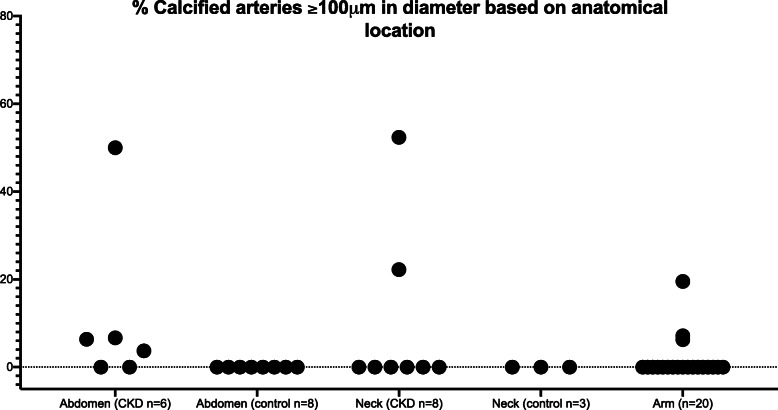
Percentage of calcified arteries ≥100 μm in diameter based on anatomical location. Each dot per column represent individual patient

**Fig. 3:**
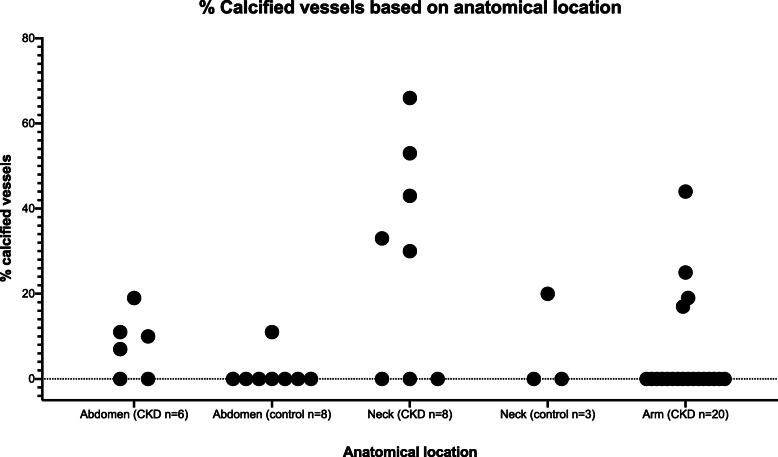
Percentage of calcified vessels in each patient based on anatomical location

Calcification was observed on von Kossa staining in small (typical diameter 50–100 μm lumen) and medium-sized (typical diameter > 100 μm lumen) vessels in 13 CKD/ESKD patients (38%) (CKD stage 4 *n* = 1, ESKD *n* = 12) and 2 control patients (18%). The difference in proportion of patients with calcification did not differ between CKD/ESKD patients and the control cohort (*p* = 0.28), however this is likely due to the small study sample size. In the majority of samples (*n* = 8), VC was predominantly localized to subcutaneous tissue, whereas in 5 samples there was an equal distribution of calcified vessels in the dermis and subcutaneous tissue. Irrespective of the tissue layer affected, VC was observed in vessels of all sizes, ranging from 50 to 200 μm in diameter. We identified more calcified vessels in neck skin samples of patients undergoing parathyroidectomy than other surgeries, which may reflect the subset of patients with severe secondary hyperparathyroidism. VC was identified at each of the three anatomical sites in patients with CKD/ESKD and in both sites sampled in the controls. Percentage of calcified vessels based on anatomical location is demonstrated in Fig. 2 (vessels > 100 μm in diameter identified as small arteries or arterioles) and Fig. 3 (all calcified vessels). The percentage of calcified vessels in this study however likely underestimates total arterial calcification since the count denominator includes veins and venules.

### VC was seen in vessel media and internal elastic lamina

**Fig. 4:**
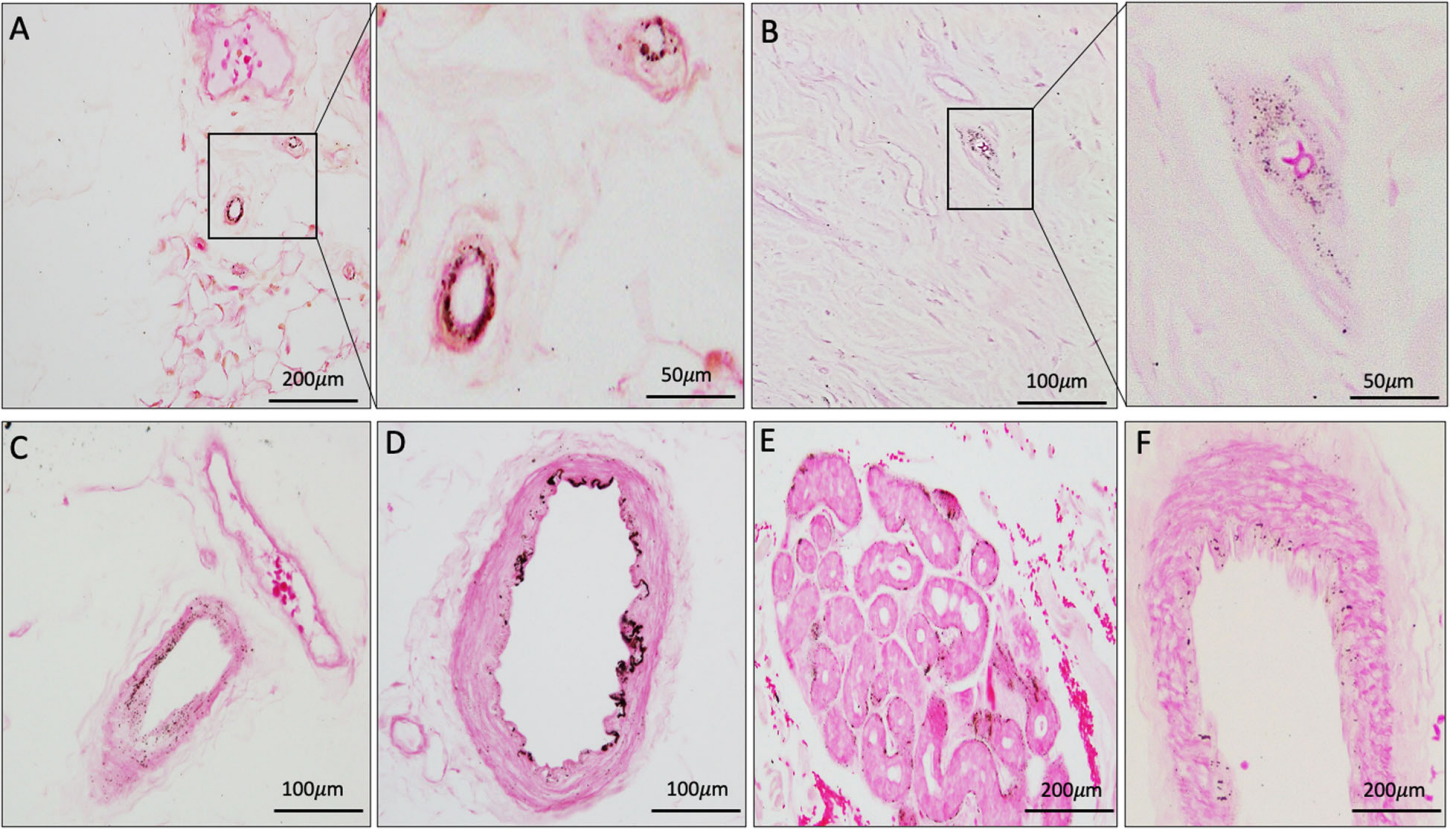
Micrographs showing various forms of calcification seen in CKD/ESKD (Fig. **a**-**e**) and control patient (Fig. **f**). Von Kossa staining shows (**a**) Vessels < 50 μm in diameter with granular and circumferential medial calcification in subcutaneous tissue and higher magnification insert of vessels highlighted in black box from Panel A. **b** Vessel 100 μm in diameter with granular calcification throughout the media and alongside the internal elastic lamina in subcutaneous tissue and higher magnification of vessel highlighted in black box from Panel B (**c**) Vessel 100 μm in diameter with granular medial calcification in dermal tissue; **d** Confluent internal elastic lamina calcification of vessel > 100 μm in diameter in subcutaneous tissue; **e** Perieccrine calcification in dermal tissue; **f** Subtle and stippled calcification of vessel > 100 μm in diameter in control patient. All photos taken at 20x magnification. Scale bar denoted in each panel

To better understand the nature of VC, we further evaluated its histological appearance and location within the vessel and whether it was granular, confluent or circumferential in nature. VC of the media was seen in patients with CKD/ESKD, with no evidence of intimal calcification. Medial VC was present as circumferential and granular calcifications in small vessels, although we were unable to determine if this involved the IEL (Fig. 4a). In larger vessels the granular calcification was noted within the vessel media and alongside the IEL (Fig. 4b), and some vessels displayed confluent areas of calcification (Fig. 4c). There was no evidence of circumferential medial calcification seen, however there was evidence of circumferential linear calcification of the IEL affecting vessels > 100 μm in diameter in two patients with ESKD (Fig. 4d).

### Concurrence of perieccrine calcification in samples with medial VC

Extra-vascular calcification was identified surrounding sweat glands (perieccrine) in 13 participants with CKD and one control participant (Fig. 4e). Of the participants with perieccrine calcification, 10 also had VC. There was no intimal hyperplasia or other pathological features consistent with calciphylaxis, including intimal thrombosis, panniculitis or hyperplasia. No other form of extra-vascular calcification was identified, including sub-cutaneous septa.

### VC in the control group was mild compared to the CKD/ESKD group

Two patients in the control group had presence of subtle and stippled medial calcification (Fig. 4f). Both patients were female, aged 65 and 71 years of age with no significant co-morbidities and normal biochemistry. One patient underwent a donor nephrectomy and had calcification present in 11% of vessels, mostly involving large arterioles. The second patient underwent a total thyroidectomy and was found to have calcification in 20% of vessels, all within subcutaneous tissue. The pattern of VC in the control group appeared distinct to that in the CKD/ESKD group with no evidence of granular or confluent calcification, or involvement of the IEL. There was evidence of perieccrine calcification in one patient in the control cohort.

### Similar demographic and biochemical markers of mineral metabolism in patients with and without skin and subcutaneous VC

In order to determine if the presence of VC was related to demographic and clinical features, we compared the specific characteristics of CKD/ESKD patients with and without VC (Table 3). Patients in both groups were of a similar age (*p* = 0.6) and dialysis vintage (*p* = 0.2). Median BMI was similar between both groups (*p* = 0.2), as was the co-morbidity profile. With respect to systemic biochemical measures of mineral metabolism and medical therapy, there were no differences in serum calcium, phosphate, ALP or PTH, or in the use of calcium- or non-calcium-based phosphate binders or activated vitamin D between groups.

**Table 3:** Differences between CKD/ESKD participants with and without VC

Demographic features	VC present (***n*** = 13)	VC absent (***n*** = 21)	***P*** value
Age, years	57 ± 13	59 ± 18	0.60
Sex (male)	8 (61%)	17 (80%)	0.21
BMI (kg/m^2^)	27 ± 4	29 ± 6	0.24
Time on dialysis (if ESKD), years	2.5 [1.77–6]	4.2 [2–6.5]	0.25
CKD stage			
Pre-dialysis	1 (eGFR 17 ml/min^2^)	2 (mean eGFR 20 ± 0.7 ml/min^2^)	
PD	4	1	
HD	8 (57%)	18 (86%)	
Diabetes	4 (30%)	9 (42%)	0.55
Hypertension	10 (77%)	14 (67%)	0.52
IHD	3 (23%)	4 (19%)	0.78
PVD	1 (7%)	3 (14%)	0.56
Smoker (current or former)	8 (57%)	15 (50%)	0.66
Previous parathyroidectomy	2 (15%)	2 (9%)	0.64
Calcitriol use	7 (50%)	10 (34%)	0.24
Non-calcium-based binder	7 (50%)	11 (38%)	0.35
Calcium based binder	2 (15%)	10 (47%)	0.06
PTH, pmol/L	58 [37–184]	66 [37–123]	0.41
Total calcium, mmol/L	2.3 ± 0.2	2.4 ± 0.2	0.57
Phosphate, mmol/L	2 ± 0.5	1.9 ± 0.7	0.71
ALP, IU/L	106 [76–207]	108 [81–139]	0.72
Vessel number	44 ± 19	39 ± 15	0.91
Perieccrine calcification	9 (69%)	4 (19%)	0.003

Results are mean ± SD, median [IQR] or number (%)

*Abbreviations*: *ALP* alkaline phosphatase, *BMI* body mass index, *HD* haemodialysis, *IHD* ischaemic heart disease, *PD* peritoneal dialysis, *PTH* parathyroid hormone, *PVD* peripheral vascular disease

### No evidence of upregulation of pro-osteogenic gene expression in patients with ESKD or with VC

**Fig. 5:**
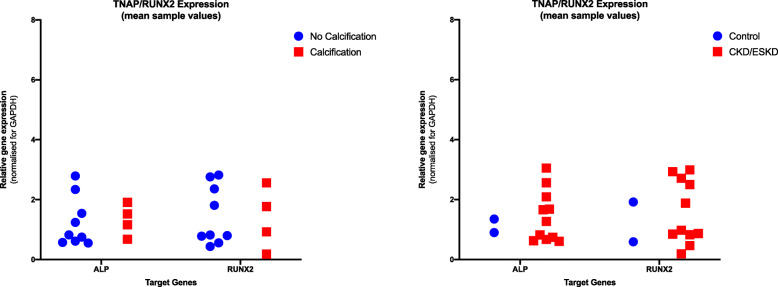
**a** Mean relative expression of *TNAP* and *RUNX2* in participants with and without histological vascular calcification; **b** Relative expression of *TNAP* and *RUNX2* in participants with CKD4/5 compared to those with normal kidney function

De novo expression of pro-osteogenic proteins has been widely implicated as a key driver in the pathogenesis of VC as well as in vascular lesions of patients with calciphylaxis [15]. Accordingly, we looked for changes in expression of two previously identified pro-calcific factors: tissue non-specific alkaline phosphatase (TNAP), which cleaves the mineralisation inhibitor pyrophosphate to phosphate, and RUNX2*,* a master transcription factor regulating osteogenic gene programs. Thirteen patients with eligible skin samples were evaluated for mRNA expression. Each skin sample was divided into three segments and tested in triplicate. Results are expressed as mean mRNA expression of each sample. There was minimal variability in gene expression across the three segments of tissue per patient sample. There was no upregulation of gene transcripts in samples with histological evidence of calcification (*n* = 4) compared to those without (*n* = 9) for either *TNAP* or *RUNX2*, *p* = 0.76 and *p* = 0.35 respectively (Fig. 5a). When comparing gene expression in patients with CKD/ESKD (*n* = 11) to those with no CKD (*n* = 2), there was also no difference in gene expression for *TNAP* or *RUNX2*, *p* = 0.8 and *p* = 0.98 respectively (Fig. 5b).

## Discussion

To our knowledge, this is the first study to show evidence of medial VC in subcutaneous and dermal vessels in multiple anatomical locations in patients with advanced CKD and ESKD undergoing elective surgery, without peripheral vascular disease or calciphylaxis. Calcification was present in 38% of samples from patients with CKD/ESKD, predominantly in the subcutaneous tissue and involving the vessel media as well as the IEL. Perieccrine calcification was identified in 64% of samples with VC, present mainly in patients with ESKD on dialysis. These microvascular calcifications were not related to biochemical markers of mineral metabolism or changes in pro-calcific gene transcripts.

Von Kossa staining is routinely used to identify tissue calcification [2]. Using this stain, two distinct types of VC were identified in our skin samples, medial calcification and calcification of the IEL. The medial calcification identified in patients with CKD/ESKD was mostly granular but becoming confluent in some of the affected vessels. Calcification identified in the two control patients was more diffuse and milder in nature, which may represent a less advanced stage of VC progression or perhaps a different pathophysiological process. There was no evidence of intimal calcification in our study and there remains conjecture as to whether medial VC and calcification of the IEL represent progression of the same disease process. Multiple studies have shown that arterial medial calcification typically starts along the IEL and in the absence of inflammation [16, 17]. In contrast, arterial intimal calcification is characterized by focal mineral deposition in highly inflamed and necrotic atherosclerotic lesions.

The high prevalence of perieccrine calcification in patients with VC is a novel finding in our study. There is little literature regarding this histological entity and its clinical significance. Mochel et al. [8] initially described perieccrine calcification in a retrospective cohort of patients with calciphylaxis, suggesting that this histological feature was highly specific to calciphylaxis - the diagnosis of calciphylaxis being made based on this histological feature alone in 4 out of 56 cases. It was also identified in a case report of calciphylaxis [18]. In a recent case series of patients with calciphylaxis (13), perieccrine calcification was not present, however this may have been due to lack of specific staining for calcification. The authors argued that given the subtleness of this histological feature, it may be possible it is routinely missed with standard H&E staining. In further case series, perieccrine calcification was identified in two biopsies in patients with low to moderate risk of calciphylaxis, and was not significantly prevalent in biopsies of patients with high clinical suspicion of calciphylaxis [7]. The clinical significance of perieccrine calcification remains unknown and it may simply reflect the severity or progression of co-existent VC.

Calcification of skin tissue is considered to be a precursor to the development of calciphylaxis and is a common finding in skin biopsies in patients with this pathology [8]. The specificity of this finding was recently questioned by Ellis and O’Neill in a study of 38 skin biopsies taken for high clinical suspicion for calciphylaxis and compared with lower limb amputation skin samples in patients with ESKD [7]. All previously documented histopathological features commonly associated with calciphylaxis, including medial calcification, intimal thrombosis and extravascular calcification, were prevalent in both high-risk skin lesions and amputation samples. Only the combination of thrombosis and medial calcification was unique to the high-risk skin biopsy group. Our study confirms that medial vascular calcification and perieccrine calcification can be present in “healthy skin” taken from three different anatomical locations, and that these features indeed are not specific to calciphylaxis. We could not identify any evidence of vessel thrombosis, likely due to the low incidence of peripheral vascular disease in our CKD/ESKD cohort. This histological finding may be more prevalent in patients with significant peripheral vascular disease rather than specific to CKD alone.

There were no significant demographic or biochemical differences in patients with CKD/ESKD with and without VC. PTH in patients with VC was similar to those without, but highly variable perhaps reflecting the variability and fluctuations in PTH, as the samples used here only capture one time point. We also identified no significant differences in serum calcium, phosphate or ALP between the two groups. However, the apparent lack of discriminating factors between the two groups may in part reflect the small sample size. It is recognised that medial VC is accelerated in patients with CKD [19]. Schlieper et al identified microcalcifications in the media of iliac arteries in uraemic patients [20] without the presence of intimal calcification or major atherosclerotic plaques. Consistent with our results, the presence of calcification was not associated with abnormalities in serum calcium, phosphate or magnesium, although PTH was not evaluated. Despite these similarities, it is uncertain to what extent VC in skin and subcutaneous tissue reflects calcification in other vascular beds and whether it is related to more systemic pro-calcific processes affecting the larger central arteries.

RUNX2, a transcription factor, is a master regulator of bone formation and is essential for bone formation and bone remodelling. Some animal studies have shown that RUNX2 expression is required for osteogenic phenotypic change in smooth muscle cells [21]. This transcription factor is also reportedly upregulated in human vascular fragments of large vascular territories where calcification is present [22]. However, evidence of osteogenesis is not a consistent finding across all studies of human VC. We did not see upregulation of *RUNX2* or *TNAP* in the subset of patients tested with histological evidence of calcification. Although osteogenic transformation of vascular smooth muscle cells is one proposed hypothesis for development of medial arterial calcification, other mechanisms have been implicated including elastin degradation, smooth muscle apoptosis and more passive biochemical processes [23]. Lack of osteogenic gene upregulation in our study may also reflect temporal aspects of calcification [6, 24] or a different underlying mechanism as a culprit. Human studies involving examination of arterial calcification in breast tissue in patients with CKD demonstrated a universal absence of staining for RUNX2 and osteocalcin in early arterial calcification [6]. A previous study identifying upregulation of osteogenic gene expression in skin was in the setting of calciphylaxis with macroscopic evidence of calcification [15]. This is not reflective of the subtle microscopic calcification identified in our participant cohort that is likely to occur in response to distinct triggers and mechanisms. On the other hand, this may be as a result of technical factors relating to the small sample size, or the sporadic nature of calcification identified on histological examination. Finally, given only a small amount of tissue was processed for PCR, small areas of active calcification may have been missed.

This study was limited by a small sample size and its cross-sectional design with a single skin incisional sample at one time point, without the ability to prospectively follow up VC progression, resolution or patient outcomes. It was not possible to obtain skin samples from control patients to match all anatomical locations and a future case-controlled study may better evaluate the impact of anatomical site on the presence of calcification. Assessment of VC burden at more central sites was also not undertaken. This study had many strengths, however, including a wide distribution of skin anatomical locations sampled as well large incisional biopsy sampling, which allowed for good interpretation of skin architecture and large number of vessels (mean of 40 vessels per slide). Also, patients had no underlying skin pathology to confound results of the study including presence of skin malignancy which is commonly associated with VC.

## Conclusion

In conclusion, this study confirms the presence of medial VC in dermal and subcutaneous tissues in patients with advanced CKD and ESKD. VC was not associated with PTH levels, however, was more likely to be present in patients undergoing parathyroidectomy. The presence of medial VC and perieccrine calcification is not a specific histological finding for calciphylaxis.

## Data Availability

Comprehensive data presented in manuscript, tables and figures. Additional data available on request from the corresponding author.

## Abbreviations

ALP: alkaline phosphatase
CKD: chronic kidney disease
ESKD: end-stage kidney disease
PTH: parathyroid hormone
VC: vascular calcification

## References

[1] Ruderman I, Holt SG, Hewitson TD (2018). Current and potential therapeutic strategies for the management of vascular calcification in patients with chronic kidney disease including those on dialysis. Semin Dial.

[2] Smith ER, Hewitson TD, Holt SG (2019). Diagnostic tests for vascular calcification. Adv Chronic Kidney Dis.

[3] Goodman WG, Goldin J, Kuizon BD (2000). Coronary-artery calcification in young adults with end-stage renal disease who are undergoing dialysis. N Engl J Med.

[4] London GM, Guerin AP, Marchais SJ (2003). Arterial media calcification in end-stage renal disease: impact on all-cause and cardiovascular mortality. Nephrol Dial Transplant.

[5] Blacher J, Guerin AP, Pannier B (2001). Arterial calcifications, arterial stiffness, and cardiovascular risk in end-stage renal disease. Hypertension.

[6] O'Neill WC, Adams AL (2014). Breast arterial calcification in chronic kidney disease: absence of smooth muscle apoptosis and osteogenic transdifferentiation. Kidney Int.

[7] Ellis CL, O'Neill WC (2018). Questionable specificity of histologic findings in calcific uremic arteriolopathy. Kidney Int.

[8] Mochel MC, Arakaki RY, Wang G (2013). Cutaneous calciphylaxis: a retrospective histopathologic evaluation. Am J Dermatopathol.

[9] Essary LR, Wick MR (2000). Cutaneous calciphylaxis. An underrecognized clinicopathologic entity. Am J Clin Pathol.

[10] Weenig RH, Sewell LD, Davis MD (2007). Calciphylaxis: natural history, risk factor analysis, and outcome. J Am Acad Dermatol.

[11] Brandenburg VM, Sinha S, Specht P (2014). Calcific uraemic arteriolopathy: a rare disease with a potentially high impact on chronic kidney disease-mineral and bone disorder. Pediatr Nephrol.

[12] Nigwekar SU (2017). Calciphylaxis. Curr Opin Nephrol Hypertens.

[13] Cerio R, Calonje E (2010). Histopathology of the skin: general principles.

[14] Vinay Kumar RSCSLR, with illustrations by James AP. Robbins basic pathology. Seventh edition. Philadelphia, PA : Saunders, [2003] ©2003, Placed Published: 2003.

[15] Kramann R, Brandenburg VM, Schurgers LJ (2013). Novel insights into osteogenesis and matrix remodelling associated with calcific uraemic arteriolopathy. Nephrol Dial Transplant.

[16] Pai A, Leaf EM, El-Abbadi M (2011). Elastin degradation and vascular smooth muscle cell phenotype change precede cell loss and arterial medial calcification in a uremic mouse model of chronic kidney disease. Am J Pathol.

[17] Shanahan CM, Cary NR, Salisbury JR (1999). Medial localization of mineralization-regulating proteins in association with Monckeberg’s sclerosis: evidence for smooth muscle cell-mediated vascular calcification. Circulation.

[18] Dookhan C, Ortega LM, Nayer A (2015). Perieccrine and pericapillary calcification in calciphylaxis. J Ren Inj Prev.

[19] Gross ML, Meyer HP, Ziebart H (2007). Calcification of coronary intima and media: immunohistochemistry, backscatter imaging, and x-ray analysis in renal and nonrenal patients. Clin J Am Soc Nephrol.

[20] Schlieper G, Aretz A, Verberckmoes SC (2010). Ultrastructural analysis of vascular calcifications in uremia. J Am Soc Nephrol.

[21] Lin ME, Chen T, Leaf EM (2015). Runx2 expression in smooth muscle cells is required for arterial medial calcification in mice. Am J Pathol.

[22] Donate-Correa J, Martín-Núñez E, Hernández-Carballo C (2019). Fibroblast growth factor 23 expression in human calcified vascular tissues. Aging.

[23] Smith ER (2016). Vascular calcification in uremia: new-age concepts about an old-age problem. Methods Mol Biol.

[24] Hortells L, Sosa C, Guillen N (2017). Identifying early pathogenic events during vascular calcification in uremic rats. Kidney Int.

